# Assisted dying practices for individuals living with frailty: prevalence and eligibility

**DOI:** 10.1093/ageing/afag275

**Published:** 2026-09-11

**Authors:** Tommaso Squeri, Christina Luk, Simon N Etkind, Annabel Price, Alex Ruck Keene, Sarah A Hopkins

**Affiliations:** West Suffolk NHS Foundation Trust, Bury Saint Edmunds, UK; School of Clinical Medicine, University of Cambridge, Cambridge, UK; Primary Care Unit, Department of Public Health and Primary Care, University of Cambridge, Cambridge, UK; Palliative Care Service, Palliative Care Service, Cambridge University Hospitals NHS Foundation Trust, Cambridge, UK; Cambridge Institute of Public Health, University of Cambridge, Cambridge, UK; Centre of Medical Law and Ethics, The Dickson Poon School of Law, King's College London, London, UK; Primary Care Unit, Department of Public Health and Primary Care, University of Cambridge, Cambridge, UK

**Keywords:** assisted dying, assisted suicide, euthanasia, frailty, law, older people

## Abstract

**Background:**

Despite the high prevalence of frailty among individuals nearing the end of life, there is uncertainty around assisted dying practices (encompassing physician-assisted suicide and voluntary euthanasia) in this population.

**Aim:**

We aimed to (i) describe the prevalence of frailty among individuals accessing assisted dying (AD), and to describe whether frailty was the main condition or a comorbidity and (ii) where prevalence data are not available, determine whether individuals are likely to be eligible for AD on the basis of their frailty.

**Methods:**

We reviewed publicly available statistics on AD usage and the relevant legal provision from jurisdictions where AD is regulated.

**Results:**

Only Canada and the Netherlands report the prevalence of frailty among individuals accessing AD. In Canada, 9.07% of deaths by AD in 2023 were associated with frailty (either as a main condition or as a comorbidity). In the Netherlands, 3.85% of deaths by AD involved multiple geriatric syndromes in 2023.

We identified 11 jurisdictions in which it would be unlikely for frailty to be an eligible condition to access AD, 6 jurisdictions where severe frailty meets or would be likely to meet eligibility criteria to access AD and 14 jurisdictions where it is unclear.

**Conclusion:**

There is a lack of detailed reporting and ongoing ambiguity about eligibility of frailty in AD across many jurisdictions. These findings support the need for improved data reporting and clarity in regulation to provide more certainty to people living with frailty and their healthcare providers.

## Key points

- Data on the prevalence of frailty in the population accessing assisted dying (AD) are only available for two jurisdictions
- In Canada, 9% of deaths by AD in 2023 were associated with frailty, either as a main condition or as a comorbidity
- In the Netherlands, 3.85% of deaths by AD in 2023 involved multiple geriatric syndromes as the primary condition
- Out of the 31 jurisdictions where AD is regulated, in 14 of these it is unclear whether frailty would be an eligible condition
- This risks causing uncertainty for those individuals with frailty who are seeking AD, and their carers and practitioners

## Introduction

Frailty is common and increasing. It is estimated to have a global prevalence of between 10% and 24% in older adults [1], and the proportion of people aged more than 60 years worldwide is expected to almost double by 2050 [2]. Despite the high prevalence of frailty among individuals nearing the end of life, there is uncertainty around assisted dying practices in this population [3].

Assisted dying (AD) is an umbrella term that refers to the regulated procedures intending to provide death at the patient’s voluntary request, subject to specified conditions [4]. This encompasses both physician assisted suicide and voluntary euthanasia. The number of jurisdictions in which AD has been decriminalised, approved in principle or legalised is increasing [5]. Across the jurisdictions where AD is permitted, differences exist between eligibility criteria, safeguards and mode of administration of lethal substances.

The British Geriatrics Society defines frailty as a chronic health condition related to ageing in which the body loses its inbuilt physiological reserves to recover from periods of ill health [6]. Whilst it is recognised that varying definitions of frailty exist, the concept of frailty has been found to be clinically useful, enabling clinicians to ‘predict the outcomes and risks of health conditions, target the delivery of evidence-based interventions, and tailor clinical management, including decisions about stressful treatments’ [7]. Frailty can often arise in the context of multimorbidity but the two are different concepts: multimorbidity refers to the concurrence of two or more overt disease diagnoses, whereas frailty is a global measure of vulnerability that looks at both clinical and subclinical impairments [8].

Importantly, the illness and dying trajectory of those living with frailty differs from the trajectories of those living with cancer, organ failure or multimorbidity [9]. These illness trajectories differ in terms of physical health as well as the psychological, social and spiritual suffering they can bring [9]. Consequently, how assisted dying is implemented in frailty is likely to differ from other patient groups [10, 11]. This is reflected in recognition that frailty presents specific challenges to legislators and practitioners responsible for legislating and implementing AD [3]. For example, prognosis in frailty is difficult to predict and this, together with ambiguity over whether frailty represents a terminal illness, can make eligibility for AD unclear [3]. This may lead to uncertainty for patients, families and practitioners, potentially causing additional distress at a time that is already emotionally charged.

It is not currently clear how many people are accessing AD with frailty. It is also unclear whether frailty is considered a legitimate reason for accessing AD, and how eligibility legislation regarding frailty varies internationally. To better understand how AD is currently implemented for individuals with frailty we aimed to (i) describe the prevalence of frailty among individuals accessing AD, and to describe whether frailty was the main condition or a comorbidity and (ii) where prevalence data are not available, determine whether individuals are likely to be eligible for AD because of their frailty.

## Methods

This study looked at all jurisdictions where AD was permitted or approved in principle until 31 January 2025, including where a law has been passed that is yet to be implemented. We took a two-step approach to describe the implementation of AD in individuals living with frailty:

(i) Analysis of routinely collected publicly available data (monitoring reports) on access to AD by adults living with frailty to ascertain the prevalence of frailty among individuals accessing AD.
(ii) Analysis of eligibility criteria set down in the relevant law regulating AD to understand whether individuals living with frailty are likely to be eligible for AD.

No ethical approval was required for this study as it only used publicly available historical data reports and legal documentation. Definitions can be found in Appendix 1 in the Supplementary  Data  section.

**Table 1:** Prevalence of AD cases associated with frailty in Canada and the Netherlands [14, 15].

Jurisdiction	Year	Total deaths by AD associated with frailty	Deaths by AD associated with frailty (% of AD deaths)	Deaths by AD with frailty as main/sole condition (% of AD deaths)^a^ ^,^ ^b^	Deaths by AD with frailty as comorbidity (% of AD deaths)	Age (median)	Female proportion (%)
Canada [14]	2023	1392	9.07^c^	0.60^c^	8.47^c^	77.6	48.8
	2022	740	5.6^d^	Not reported	Not reported	77	48.6
	2021	Not reported	Not reported	Not reported	Not reported	76.3	47.7
	2020	Not reported	‘Nearly 3%’^e^	Not reported	Not reported^e^	75.3	48.1
	2019	Not reported	Not reported^f^	Not reported	Not reported	75.2	49.1
Netherlands [15]	2023	349	3.85	3.85	Not reported	Not reported^g^	49.2
	2022	379	4.35	4.35	Not reported	Not reported	49.4
	2021	307	4.00	4.00	Not reported	Not reported	50.1
	2020	235	3.39	3.39	Not reported	Not reported	48.7
	2019	172	2.70	2.70	Not reported	Not reported	48.0
	2018	205	3.35	3.35	Not reported	Not reported	47.9
	2017	293	4.45	4.45	Not reported	Not reported	48.6
	2016	244	4.01	4.01	Not reported	Not reported	49.0
	2015	183	3.32	3.32	Not reported	Not reported	Not reported
	2014	257	4.84	4.84	Not reported	Not reported	Not reported
	2013	251	5.20	5.20	Not reported	Not reported	Not reported

^a^(Canada) Frailty data are unavailable before 2019; other data remain available from 2016 onward.

^b^(Netherlands) Frailty data are unavailable before 2013; other data remain available from 2002 onward. Multiple geriatric syndromes as indicator of frailty.

^c^Frailty was reported as a medical condition in 1392 MAID cases in 2023; in 92 of these, frailty was the only reported medical condition.

^d^Frailty-associated AD cases were reported in 2022 but were not separately identified as a main condition or as a distinct comorbidity. This number is derived from reported data: 22.6% of MAID cases classified as ‘other medical condition / multiple comorbidities,’ of which 25% involved frailty.

^e^Frailty was cited as nearly 3% of MAID cases within the ‘Other Conditions and Multiple Co-morbidities’ category; it was not reported separately as Main Condition versus comorbidity.

^f^Frailty was refenced descriptively as ‘commonly cited’ in the ‘Other Conditions’ category; quantitative data not provided.

^g^Only age ranges were provided (e.g. minor to 104, minor to over 90).

### Prevalence of frailty among individuals accessing AD

#### Data identification

We examined online official AD reports published by government sources or, for Switzerland, the non-profit organisation DIGNITAS which collates official government data, from 1 January 2000–31 March 2025. Other non-governmental AD providers in Switzerland were not included due to limited data (see Appendix 2 in the Supplementary  Data section for further details). We recorded which jurisdictions did not issue any official data (see Appendix 3 in the Supplementary  Data section for further details). No language restrictions were applied. We excluded editorials, news articles, non-governmental reports and research papers. Data about access to AD in general were excluded unless frailty was mentioned. The total number of deaths in each jurisdiction was collected from government-issued reports to allow prevalence calculations.

#### Analysis

Descriptive statistics were used to summarise patterns of access and the characteristics of AD cases in jurisdictions reporting the prevalence of frailty. For jurisdictions providing individual-level patient data, mean demographic values (age, proportions by sex) were manually calculated. For jurisdictions reporting only aggregated data, the corresponding statistics were extracted as reported. Where available, the following variables were described:

- Year of data
- Number of AD deaths associated with frailty, including proxy measures such as multiple geriatric syndromes (MGS)
- Frailty-associated AD deaths as a percentage of all AD deaths
- Age and sex of applicants and decedents

### Eligibility of individuals living with frailty to access AD

#### Document identification

We identified relevant legal provisions regulating AD across 31 jurisdictions using web searches. We included only primary legislation, except for the six jurisdictions where AD is regulated by criminal codes and supreme court rulings (see Appendix 4 in the Supplementary  Data section for further details).

#### Analysis

We extracted the eligibility criteria and definitions of key terminology from each document. In those jurisdictions where no official English version of the relevant legal provision was provided, documents were translated using the in-built server translator. Translations were then reviewed and revised by a native-speaking clinician with relevant clinical experience.

We identified three categories of eligibility criteria:

(i) presence of a terminal disease or illness or condition;
(ii) presence of a terminal disease or illness or condition with unbearable suffering and
(iii) presence of unbearable suffering in or out of the context of a disease or illness or condition.

We then established which approach is adopted by each jurisdiction. Switzerland and Germany could not be categorised as their regulation of AD does not involve medical criteria.

Subsequently, we determined which form of AD is permitted in each jurisdiction (i.e. assisted suicide and/or voluntary euthanasia). For jurisdictions in which voluntary euthanasia is permitted, we also described whether advance directives are available to access it.

Finally, we made a judgement as to whether it was likely, unlikely or unclear whether the presence of severe or very severe frailty (Clinical Frailty Scale 7 or 8) would satisfy the eligibility threshold set out in each jurisdiction [12]. The multidisciplinary research team (combining experience in law, geriatrics, psychiatry, general medicine and palliative care) did so by using the definition of frailty formulated by the British Geriatrics Society [6] and their own clinical experience. Given evidence that individuals living with frailty experience pain and emotional distress at levels similar to people with cancer and evidence for multidimensional suffering in individuals living with frailty, the research team judged that severe or very severe frailty could meet a threshold of unbearable suffering, where this was present [11, 13].

We tabulated the results of our analysis to identify similarities and differences between jurisdictions, including quotations of key aspects of eligibility criteria and the reasoning for the authors’ judgements.

## Results

### Prevalence of frailty among individuals accessing AD

Of the 31 jurisdictions analysed, two jurisdictions (Canada and the Netherlands) reported data on the prevalence of frailty or related geriatric syndromes among individuals accessing AD. More commonly jurisdictions reported demographic characteristics, including age.

#### Frailty in AD data

Canada reported frailty explicitly within Medical Assistance in Dying (MAID) statistics. In the Netherlands, we considered MGS as a proxy for frailty given the definition described in its legislation (see Appendix 1 in the Supplementary  Data section).

In Canada, the proportion of AD deaths associated with frailty (as either the main condition or a comorbidity) rose from nearly 3% in 2020 to 9.1% in 2023 (Table 1). In 2023, Canada reported AD deaths with frailty as the main condition, and frailty as a co-morbidity separately. Most frailty-associated cases in 2023 (93.4%) were recorded as comorbidities rather than the main/sole condition. In all cases where frailty was the sole medical condition in 2023, most individuals were 85 years or older.

In the Netherlands, MGS represented a small but consistent subgroup of AD cases. Unlike Canada, in the Netherlands MGS is reported only when it constitutes the primary condition and is not captured as a comorbidity. The highest proportion (5.2%) was recorded in 2013, with more recent years (2019–2023) fluctuating between 2.7% and 4.35%.

#### Age of individuals accessing AD

The prevalence of frailty is strongly correlated with age, although they are not synonymous. Among the 22 jurisdictions with publicly available AD reports, nine reported average ages (mean or median) for patients who died by AD, and four reported average ages for patients who requested AD. The remaining jurisdictions either did not report age, provided only an age range, or presented general statistics (e.g. the proportion of patients over 60 years). These jurisdictions were excluded from Table 2.

**Table 2:** Average age of patients who died by or requested AD across reporting years in jurisdictions with available data [14, 16–26].

Jurisdiction	Data available from^a^	Age measure (mean or median)	Average age at assisted death/ at request (across reporting years)^b^
Canada	2019–2023	Mean or median	76
California (USA)	2016–2023	Mean	76
Colorado (USA)	2017–2023	Median	73
District of Columbia (USA)	2017–2022	Mean	74^c^
Hawaii (USA)	2019–2023	Mean	75
New Jersey (USA)	2019–2023	Mean	73
Oregon (USA)	2000–2024	Mean	72
Spain	2021, 2022–2023	Median	65 (F), 66 (M) 2022: 65 (F), 66 (M)^e^ 2023: 78^e^
Tasmania (Australia)	2022–2024	Average, unspecified	73^e^
Victoria (Australia)	2024	Average, unspecified	73^e^
Washington (USA)	2019–2023^d^	Did not specify	74
Western Australia (Australia)	2022–2024	Median	74^e^

^a^In Australian jurisdictions, reporting is based on financial years, e.g. 2022 corresponds to 1 July 2021 to 30 June 2022.

^b^Rounded to the nearest integer

^c^No AD deaths in 2017 in District of Columbia.

^d^Only age ranges reported from 2009–2018.

^e^Average age at AD request (not age at assisted death)

All jurisdictions reporting data had average ages of 65 years or older. Where age data were available for AD requests, reported average or median ages were similarly high, ranging from 73 to 78.5 years.

### Eligibility of individuals living with frailty to access AD

We identified 11 jurisdictions that adopted an approach requiring the presence of terminal disease or illness or condition in their eligibility criteria for AD (Table 3) and 10 jurisdictions that adopted an approach requiring the presence of disease or illness or condition with unbearable suffering (Table 4). Eight jurisdictions adopted an approach requiring the presence of unbearable suffering in or out of the context of a disease or illness or condition (Table 5).

**Table 3:** Jurisdictions adopting medical criteria for eligibility (i) presence of a terminal disease or illness or condition.

Jurisdiction	Medical criteria for eligibility	Prognostic criteria	Forms of AD available	Criteria or definition that frailty would have to meet for eligibility	Likelihood of frailty meeting the criteria for eligibility
California (USA)	Terminal disease	<6 months	Assisted suicide	‘incurable and irreversible disease that has been medically confirmed and will […] result in death within six months’ [32]	Unlikely—frailty is unlikely to be considered a disease
Colorado (USA)	Terminal disease	<6 months	Assisted suicide	‘incurable and irreversible disease that has been medically confirmed and will […] result in death within six months’ [33]	Unlikely—frailty is unlikely to be considered a disease
District of Columbia (USA)	Terminal disease	<6 months	Assisted suicide	‘incurable and irreversible disease that has been medically confirmed and will […] result in death within 6 months’ [34]	Unlikely—frailty is unlikely to be considered a disease
Hawaii (USA)	Terminal disease	<6 months	Assisted suicide	‘incurable and irreversible disease that has been medically confirmed and will […] produce within six months. […] does not include age or any physical disability or condition that is not likely to, by itself, cause death within six months’ [35]	Unlikely—frailty is unlikely to be considered a disease
Maine (USA)	Terminal disease	<6 months	Assisted suicide	‘incurable and irreversible disease that has been medically confirmed and will […] produce death within 6 months’ [36]	Unlikely—frailty is unlikely to be considered a disease
Montana (USA)^a^	Terminal illness	Short period of time	Assisted suicide	‘incurably ill and face death within a relatively short period of time’ [37]	Unclear—it is uncertain whether frailty can be considered an illness and it is difficult to predict a prognosis
New Jersey (USA)	Terminal condition	<6 months	Assisted suicide	‘terminal stage of an irreversibly fatal illness, disease, or condition with a prognosis […] of six months or less’ [38]	Unclear—frailty can be considered an irreversibly fatal condition but it is difficult to predict a prognosis
New Mexico (USA)	Terminal condition	<6 months	Assisted suicide	‘disease or condition that is incurable and irreversible and that […] will result in death within six months’ [39]	Unclear—frailty can be considered an incurable and irreversible condition but it is difficult to predict a prognosis
Oregon (USA)	Terminal disease	<6 months	Assisted suicide	‘incurable and irreversible disease that has been medically confirmed and will […] produce death within six months’ [40]	Unlikely—frailty is unlikely to be considered a disease
Vermont (USA)	Terminal disease	<6 months	Assisted suicide	‘incurable and irreversible disease which would […] result in death within six months’ [41]	Unlikely—frailty is unlikely to be considered a disease
Washington (USA)	Terminal disease	<6 months	Assisted suicide	‘incurable and irreversible disease that has been medically confirmed and will […] produce death within six months’ [42]	Unlikely—frailty is unlikely to be considered a disease

Please note that the table provides only excerpts relevant to the study, not the entire text of each jurisdiction’s eligibility criteria

^a^AD in Montana is not regulated by statute at the time of writing of this article but is protected by a 2009 state Supreme Court ruling [37].

**Table 4:** Jurisdictions adopting medical criteria for eligibility (ii) presence of a terminal disease, illness or condition with unbearable suffering.

Jurisdiction	Medical criteria for eligibility	Prognostic criteria	Forms of AD available	Option for advance directive in AD available?	Criteria or definition that frailty would have to meet for eligibility	Likelihood of frailty meeting the criteria for eligibility
Australian Capital Territory (Australia)^a^	Terminal condition WITH unbearable suffering	Not present	Assisted suicide AND voluntary euthanasia	No	‘have been diagnosed with a condition that […] is advanced, progressive and expected to cause death and they are suffering intolerably’ [43]^a^	Likely—frailty can be considered an advanced and progressive condition and the law explicitly allows eligibility of individuals approaching the end of their lives even if the prognosis is unclear
Italy^b^	Terminal illness WITH unbearable suffering	Not present	Assisted suicide		‘kept alive by life-support treatments and suffering from an incurable illness which is a source of physical or psychological suffering that he or she considers intolerable’ [44]	Unlikely—life-support treatments are unlikely to be relevant for people living with frailty at the end of life
New South Wales (Australia)	Terminal condition WITH unbearable suffering	<6 months (<12 months for a neurodegenerative condition)	Assisted suicide AND voluntary euthanasia	No	‘disease, illness or medical condition that is advanced, progressive and will […] cause death within a period of 6 months […] and is causing suffering to the person that cannot be relieved in a way that person considers tolerable’ [45]	Unclear—frailty can be considered an advanced and progressive condition but it is difficult to predict prognosis
New Zealand	Terminal illness WITH unbearable suffering	<6 months	Assisted suicide AND voluntary euthanasia	No	‘terminal illness that is likely to end the person’s life within 6 months and’ is in advanced state of irreversible decline in physical capability AND ‘experiences unbearable suffering’ [46]	Unclear—it is uncertain whether frailty can be considered an illness and it is difficult to predict a prognosis
Portugal^c^	Terminal disease WITH unbearable suffering	Not present	Assisted suicide AND voluntary euthanasia	Not present	‘serious, permanent, and widely disabling injury that places the person in a situation of dependence on a third party […] for carrying out basic activities of daily living, with […] very high probability that such limitations will persist over time without the possibility of […] significant improvement’ OR ‘a life-threatening disease in an advanced and progressive stage, incurable and irreversible, and that causes intense suffering’ [47]	Unlikely—frailty is unlikely to be considered an injury or disease
Queensland (Australia)	Terminal condition WITH unbearable suffering	<12 months	Assisted suicide AND voluntary euthanasia	No	‘disease, illness or medical condition that is advanced, progressive and will cause death and is expected to cause death within 12 months and is causing suffering that the person considers to be tolerable’ [48]	Unclear—frailty can be considered an advanced and progressive condition but it is difficult to predict prognosis
South Australia (Australia)	Terminal condition WITH unbearable suffering	<6 months (<12 months for a neurodegenerative condition)	Assisted suicide AND voluntary euthanasia	No	‘disease, illness or medical condition that is incurable and is advanced, progressive and will cause death and is expected to cause death […] not exceeding 6 months and is causing suffering to the person that cannot be relieved in a manner that the person considers tolerable’ [49]	Unclear—frailty can be considered an incurable, advanced and progressive condition but it is difficult to predict prognosis
Tasmania (Australia)	Terminal condition WITH unbearable suffering	<6 months (<12 months for a neurodegenerative condition)	Assisted suicide AND voluntary euthanasia	No	‘disease, illness, injury, or medical condition, of the person that is advanced, incurable and irreversible, and […] is expected to cause the death of the person and is expected to cause the death of the person within 6 months’ [50]	Unclear—frailty can be considered an advanced, incurable and irreversible condition but it is difficult to predict prognosis
Victoria (Australia)	Terminal condition WITH unbearable suffering	<6 months (<12 months for a neurodegenerative condition)	Assisted suicide AND voluntary euthanasia	No	‘disease, illness or medical condition that is incurable and is advanced, progressive and will cause death and is expected to cause death […] not exceeding 6 months and is causing suffering to the person that cannot be relieved in a manner that the person considers tolerable’ [51]	Unclear—frailty can be considered an incurable, advanced and progressive condition but it is difficult to predict prognosis
Western Australia (Australia)	Terminal condition WITH unbearable suffering	<6 months (<12 months for a neurodegenerative condition)	Assisted suicide AND voluntary euthanasia	No	‘disease, illness or medical condition that is advanced, progressive and will cause death and will […] cause death within a period of 6 months and […] is causing suffering to the person that cannot be relieved in a manner that the person considers tolerable’ [52]	Unclear—frailty can be considered an advanced and progressive condition but it is difficult to predict prognosis

Please note that the table provides only excerpts relevant to the study, not the entire text of each jurisdiction’s eligibility criteria.

^a^The Voluntary Assisted Dying Act 2024 regulating AD in Australian Capital Territory was passed in June 2024 but will not be operating until November 2025.

^b^AD in Italy is no regulated by national statute at the time of writing of this article but is protected by a 2019 Constitutional Court ruling [44].

^c^The Lei n°22/2023 regulating AD in Portugal was passed in May 2023 but it is not operative at the time of writing of this article due to ongoing constitutional reviews and regulatory delays.

**Table 5:** Jurisdictions adopting medical criteria for eligibility (iii) presence of unbearable suffering in or out of the context of a disease or illness or condition.

Jurisdiction	Medical criteria for eligibility	Prognostic criteria	Forms of AD available	Option for advance directive in AD available?	Criteria or definition that frailty would have to meet for eligibility	Likelihood of frailty meeting the criteria for eligibility
Austria	Terminal illness OR presence of unbearable suffering in the context of an illness	Not present	Assisted suicide		‘incurable, fatal illness’ or ‘a serious, permanent illness with persistent symptoms, the consequence of which permanently impaired the affected person’s entire lifestyle where the illness causes suffering that cannot be averted by any other means’ [53] illness is defined as ‘an abnormal physical or mental state that necessitates medical treatment’ [54]	Unclear—difficult to establish whether an abnormal physical state necessitating medical treatment would apply to frailty
Belgium	Unbearable suffering the context of a condition	Not present	Voluntary euthanasia	Yes	‘hopeless medical situation and reports constant and unbearable physical or mental suffering which cannot be relieved and which results from a serious and incurable accidental or pathological condition’ [55]	Unclear—difficult to establish whether a hopeless medical situation would apply to frailty
Canada	Unbearable suffering in the context of an illness	Not present	Assisted suicide AND voluntary euthanasia	No	‘serious and incurable illness, disease or disability’ and ‘an advanced state of irreversible decline in capability’ AND ‘that illness, disease or disability or that state of decline causes them enduring physical or psychological suffering that is intolerable to them and that cannot be relieved under conditions that they consider acceptable’ [56]	Already eligible
Colombia^a^^,^^b^	Unbearable suffering in the context of an illness	Not present	Assisted suicide AND voluntary euthanasia	Yes	‘intense physical or psychological suffering, resulting from bodily injury or serious and incurable illness’ [57]	Unclear—it is uncertain whether frailty can be considered an illness
Ecuador^b^^,^^c^	Unbearable suffering in the context of a disease	Not present	Voluntary euthanasia		‘intense suffering resulting from an injury that is necessarily bodily, serious and irreversible, or a disease that is serious and incurable’ [58]	Unlikely—frailty is unlikely to be considered an injury or disease
Luxembourg	Unbearable suffering in the context of a condition and hopeless medical situation	Not present	Assisted suicide AND voluntary euthanasia	Yes	‘hopeless medical situation and […] constant and unbearable physical or psychological suffering without prospect of improvement, resulting from an accidental or pathological condition’ [59]	Unclear—difficult to establish whether a hopeless medical situation would apply to frailty
The Netherlands	Unbearable suffering	Not present	Assisted suicide AND voluntary euthanasia	Yes	‘the physician […] has become convinced that the patient was suffering from hopeless and unbearable pain’ [60]	Already eligible as ‘multiple geriatric syndrome’
Spain	Unbearable suffering in the context of a condition or disease	Not present	Assisted suicide AND voluntary euthanasia	Yes	‘serious, chronic, and disabling condition: […] limitations that directly affect physical autonomy and activities of daily living, preventing the individual form being self-reliant, as well as their capacity for expression and interaction, and which are associated with constant and intolerable physical or psychic suffering for the person experiencing it. There is certainty or a high probability that these limitations will persist over time without the possibility of a cure or appreciable improvement. Sometimes it can mean absolute dependence of technological support’ OR ‘serious and incurable disease: […] causes constant and unbearable physical or psychic suffering without the possibility of relief which the person considers tolerable, with a limited life expectancy, in a context of progressive frailty’ [61]	Likely—frailty can be considered a serious, chronic and disabling condition with the limitations described

Please note that the table provides only excerpts relevant to the study, not the entire text of each jurisdiction’s eligibility criteria

^a^Constitutional Court Ruling C-233/21 [62] and Constitutional Court Ruling C-164/22 [57] eliminated the requirement for a terminal illness and decriminalise medically assisted suicide respectively. This contrasts with the Colombia’s Ministry of Health and Social Protection Resolution 971 of 2021 (the most recent one regulating this area), which maintains the criteria for a terminal illness and regulated voluntary euthanasia but does not provide for assisted suicide.

^b^The native-speaking clinician who reviewed and revised the translations for Colombia and Ecuador, translated the term *enfermedad*, which is found in both jurisdictions’ provisions, into illness for Colombia and into disease for Ecuador.

^c^AD in Ecuador is not regulated by statute at the time of writing of this article but is protected by a 2024 Constitutional Court ruling [58].

Switzerland and Germany could not be categorised according to the three approaches described.

- In Switzerland, assisted suicide is provided on the basis of Article 115 of the Swiss Penal Code, which condones assisting suicide if it is not motivated by selfish motives [27] (see Appendix 2  in  the  Supplementary  Data  section  for  further  details).
- In Germany, the Federal Constitutional Court approved the permissibility of assisting in suicide in principle, highlighting that this may not be linked to *substantive criteria,* including medical criteria [28].

Of the 11 jurisdictions that define medical eligibility on the presence of a terminal disease or illness, we found that frailty would be unlikely to be an eligible condition in eight of them (California, Colorado, District of Columbia, Hawaii, Maine, Oregon, Vermont, Washington) because eligibility requires the presence of an ‘incurable and irreversible disease’ (Table 3). The authors judged that it is unlikely that frailty would meet this criterion, as frailty tends to be considered a condition rather than a disease [29].

There were two jurisdictions (New Jersey, New Mexico) which based eligibility on the term ‘condition’ and Montana which said ‘incurably ill’ (Table 3). However, all three have a prognostic requirement for eligibility (‘less than 6 months’ in two, ‘relatively short period of time’ in one) and, given the uncertainty around clinical trajectories in frailty [13, 30, 31], the authors felt it unclear whether frailty would meet the criteria.

Of the 10 jurisdictions that define medical eligibility on the presence of a terminal disease, illness or condition with unbearable suffering, we considered it unclear whether frailty would be eligible to access AD in seven of them (New South Wales, New Zealand, Queensland, South Australia, Tasmania, Victoria, Western Australia), as they all require a prognosis of <6 or <12 months (Table 4).

There were two jurisdictions (Italy, Portugal) where we considered it unlikely that frailty would meet eligibility, as these require the presence of ‘life-support treatments’ and an ‘injury or disease’ respectively.

In Australian Capital Territory, it is likely that frailty would be eligible as the term ‘condition’ is included and no prognostic requirement is mentioned besides the requirement for an individual to be approaching the end of their life. The law here specifically states that an individual may be approaching the end of their life even if it is uncertain whether the condition will cause death within the next 12 months [43].

Of the eight jurisdictions that define medical eligibility on the presence of unbearable suffering in or out of the context of a disease or illness or condition (Table 5), frailty is already eligible in two of these (Canada, Netherlands). We also judged that the presence of the term ‘condition’ without prognostic requirements make it likely for frailty to be eligible in Spain.

In two jurisdictions (Belgium, Luxembourg), the requirement of a ‘hopeless medical situation’ make the eligibility of frailty unclear as the term ‘hopeless’ is difficult to interpret clinically. This is also the case for Austria and Colombia, where it is uncertain whether frailty would be considered an ‘illness’. In Ecuador, we judged that frailty is unlikely to be eligible as it requires the presence of a ‘disease’.

Switzerland and Germany separate eligibility to AD from medical criteria. Switzerland decriminalises the assistance to suicide if conducted without *selfish motives* [27]. The German federal constitutional court does not link the permissibility of suicide assistance to a substantive criterion such as requiring a diagnosis of incurable or terminal illness [28]. Given the absence of medical criteria for eligibility, we judged it likely that frailty would be eligible in Switzerland and Germany.

## Discussion

Only two out of 31 jurisdictions reported on the prevalence of frailty or MGS in those accessing AD. Consequently, the prevalence of frailty among those accessing AD is largely unknown. There is ambiguity and variation internationally regarding whether frailty constitutes an eligible condition for access to AD. There are six jurisdictions in which frailty already meets or is likely to meet eligibility criteria to access AD, 11 jurisdictions in which it is unlikely that frailty is an eligible condition to access AD, and 14 jurisdictions where this is unclear. Overall, frailty was less frequently judged to be eligible where there is a strict prognostic or terminal disease criterion. Uncertainty over eligibility arises from two main sources: how prognostic criteria are implemented, and how the terms found in the law are interpreted.

Previous research has analysed the prevalence of underlying diseases leading to AD deaths, and found cancer to be the most common condition at 66.5% of AD recipients, followed by nervous system diseases at 8.1% [63]. To our knowledge, ours is the first study to look at the prevalence of frailty, and the results suggest it is an important reason for accessing AD within the two jurisdictions for which public data are available. Understanding assisted dying practices in frailty is important given that these are likely to differ from other eligible groups, as well as the high prevalence of frailty among those nearing the end of life [64]. The current lack of clarity can lead to uncertainty for patients, families and practitioners: a better understanding of the issues facing those seeking AD who live with frailty may help to reduce this uncertainty. Further research is needed to understand delegated legislation (such as codes of practice) and the lived experiences of those with frailty wanting to access AD. Contacting individual regulators to request access to assessment reports of individuals requesting access to AD on the basis of frailty may also further our understanding.

Murray and Etkind highlighted that using prognostic criteria to assess eligibility for AD leads to ambiguity [65]. Our results show that this is particularly the case of conditions with uncertain disease trajectories such as frailty [66]. Among the 31 jurisdictions included, there were 10 where prognostic criteria contributed to making it unclear whether frailty would be an eligible condition. This raises questions regarding whether prognostic uncertainty around the end of life in people living with frailty can lead to a form of ‘disadvantaged dying’, where individuals are not deemed eligible to access to AD because of these clinical limitations [65, 67, 68].

Our findings show that uncertainty over eligibility also arises because of the ambiguity of key terminology in the law: this was the case in five jurisdictions. For example, the distinction between terms like *disease* and *illness* has long been a source of debate [69]. Lach argues that, from a clinical perspective, a *disease* is usually seen as an ‘objective’ entity afflicting a patient and, by contrast, *illness* as the subjective experience of suffering [69]. Following this interpretation, it would be unlikely that frailty would be considered a *disease* as this usually requires a pathological evaluation (i.e. chemical or biological markers) [69], which is not necessary in the identification of frailty [70]. Where access to AD is based on presence of disease, this may exclude those dying with frailty. In those jurisdictions where *illness* is used, the interpretation is more ambiguous. If considering the term *illness* as only the subjective experience of the patient, frailty may be an eligible condition. However, *illness* can also imply the presence of a *disease,* which causes the patient to be *ill* [69]. In this latter interpretation, individuals would still need to suffer from a *disease* to be considered *ill,* making frailty unlikely to be a sufficient condition. The complex application of legal terminology to the clinical context is also encountered in jurisdictions where English is not the official language. For example, the Spanish term *enfermedad* does not draw a clear distinction between the ‘objective’ entity and the subjective experience of the sufferer [71], making it therefore difficult to know whether frailty would be considered an *enfermedad*.

### Strengths and limitations

We accessed relevant legal documents in all 31 jurisdictions and data on AD use in all 22 jurisdictions where such data are available, making this a comprehensive summary of publicly available data. The multidisciplinary research team included clinicians from a range of specialities, as well as a lawyer. However, this study has limitations. Whilst we investigated primary legislation, criminal codes and supreme court rulings concerning AD, delegated legislation such as codes of practices or national and local guidelines were not analysed as they were not easily comparable between different jurisdictions. Further research should investigate whether these documents resolve ambiguities about the position of frailty in AD legislation.

In Austria, Belgium, Colombia, Ecuador, Luxembourg, Netherlands and Portugal, the documents regulating AD are only available in the jurisdictions’ respective official languages. The in-built server translator was initially used to translate these. The translations were then reviewed and revised by native-speaking clinicians with relevant clinical experience, to improve accuracy and aid interpretation. We cannot exclude the possibility that some of the nuances of legal terms have been lost in the translation process. Moreover, the judgements made by the authors on the likelihood of frailty being an eligible condition to access to AD in the jurisdictions examined are interpretations based on their understanding of the terminology used and own medical knowledge and experience. There may be differences in how frailty is conceptualised in the different jurisdictions, which is a limitation of our findings. We have, however, presented relevant quotations from each jurisdiction and the rationale for our eligibility interpretations, allowing readers to review and assess the basis for each decision. That others might reach different conclusions serves to highlight the challenges for those of judging eligibility in applying the law in their respective jurisdictions.

## Conclusion

Whilst there is a high prevalence of frailty among individuals nearing the end of life, frailty has not been well considered when designing eligibility criteria for AD in many jurisdictions. This is reflected in ambiguity over the eligibility of those living with frailty to access AD, and the lack of public reporting on the prevalence of frailty among those accessing AD. This lack of clarity risks causing uncertainty for those individuals with frailty who are seeking AD, their carers and clinicians involved. Clearer reporting on the clinical characteristics of individuals accessing AD would help to address this and provide additional information to individuals wishing to access AD, their families and clinicians.

## Supplementary Material

aa-26-1171-File002_afag275
