# Supplementary material for: Assisted dying practices for individuals living with frailty: prevalence and eligibility
Source: Age Ageing. 2026 Sep 11;55(9):afag275. doi: 10.1093/ageing/afag275 (PMC13565127; doi:10.1093/ageing/afag275)
Supplement: Supplementary file 1 [file aa-26-1171-file002_afag275.docx]

**Assisted dying practices for individuals living with frailty: prevalence and eligibility**

**Contents**

Appendix 1: List of definitions

Appendix 2: Additional details of data reporting and legal situation regarding AD in Switzerland

Appendix 3: Jurisdictions with and without publicly accessible AD reports

Appendix 4: Type of legal document regulating AD in each jurisdiction

**Appendix 1**

**List of definitions**

Advance directive**:** a legal document that allows a mentally competent individual to outline his preference regarding specified medical decisions that he may not be able to make in the future [73]

Assisted dying: an umbrella term that refers to the regulated procedures intending to terminate a person’s life at the person’s voluntary request, subject to specified conditions [4]

Multiple geriatric syndromes: These syndromes, which are often degenerative in nature, generally occur in elderly patients, and a combination of these syndromes and the related symptoms can cause suffering. Examples including sight impairment, hearing impairment, osteoporosis, osteoarthritis, balance problems, or cognitive deterioration that may cause unbearable suffering without prospect of improvement [74]

Physician assisted suicide**:** the regulated assistance of a physician to terminate a person’s life by providing a drug for self-administration at the person’s voluntary request [4]

Voluntary euthanasia: the regulated administration by a physician or a third person of a drug with the specific intent to terminate the person’s life at the person’s voluntary request [4]

Jurisdiction: the limits or territory within which legal authority may be exercised

Frailty: a chronic health condition related to ageing in which the body loses its inbuilt physiological reserves to recover from periods of ill health [6]

Primary legislation: the laws that are enacted by the main legislative body, such as a parliament

**Appendix 2**

**Additional details of data reporting and legal situation regarding AD in Switzerland**

There are a number of non-governmental AD providers in Switzerland. These organisations vary in the extent to which they publish data relevant to this study.

Organisations with available data:

- DIGNITAS: Publishes annual statistics on assisted dying. However, no data specific to frailty or frailty-related conditions were identified.
- EXIT ADMD (Suisse romande, French-speaking Switzerland): Published reports are available from 2011 to 2018. These include data on assisted deaths involving “disabling polypathologies” [75]. However, as it covers only a small part of the jurisdiction, it was not included in the main analysis.

Organisations without publicly available data:

- EXIT (Deutsche Schweiz, German-speaking Switzerland)
- Pegasos Swiss Association
- Lifecircle / Eternal Spirit

Newly established organisations with no publicly available data at the time of analysis:

- Athanasios
- Phoenix Care

Other:

- The Last Resort: not currently accepting applications; legal status under challenge. No data available.

Three cantons (Geneva, Neuchâtel, Vaud) have cantonal statute laws setting out medical criteria for access within medical and medico-social institutions [76-78].

**Appendix 3**

**Jurisdictions with and without publicly accessible AD reports**

| *Reports accessible* | *No reports published* |
| --- | --- |
| Australia – New South Wales | Australian Capital Territory^1^ |
| Queensland | Austria |
| South Australia | Colombia |
| Tasmania | Ecuador |
| Victoria | Germany |
| Western Australia | Italy |
| Belgium | Portugal^2^ |
| Canada | Montana |
| Luxembourg | New Mexico |
| Netherlands |  |
| New Zealand |  |
| Switzerland (limited) |  |
| US - California |  |
| Colorado |  |
| District of Colombia |  |
| Hawaii |  |
| Maine |  |
| New Jersey |  |
| Oregon |  |
| Vermont (limited) |  |
| Washington |  |
| Spain |  |

^1^AD in Australian Capital Territory is not operative at the time of writing

^2^AD in Portugal is not operative at the time of writing

**Appendix 4**

**Type of legal document regulating AD in each jurisdiction**

| ***Jurisdiction*** | ***Forms of AD available*** | ***Type of legal document currently regulating AD*** |
| --- | --- | --- |
| Australian Capital Territory | Assisted suicide AND voluntary euthanasia | State parliament act |
| Austria | Assisted suicide | Federal parliament act |
| Belgium | Voluntary euthanasia | Federal parliament act |
| Canada | Assisted suicide AND voluntary euthanasia | Federal parliament act |
| California (USA) | Assisted suicide | State legislature act |
| Colombia | Assisted suicide AND voluntary euthanasia | National constitutional court ruling |
| Colorado (USA) | Assisted suicide | State legislature act |
| District of Columbia (USA) | Assisted suicide | District council act |
| Ecuador | Voluntary euthanasia | National constitutional court ruling |
| Germany | Assisted suicide | Federal constitutional court ruling |
| Hawaii (USA) | Assisted suicide | State legislature act |
| Italy | Assisted suicide | National constitutional court ruling |
| Luxembourg | Assisted suicide AND voluntary euthanasia | National parliament act |
| Maine (USA) | Assisted suicide | State legislature act |
| Montana (USA) | Assisted suicide | State supreme court ruling |
| The Netherlands | Assisted suicide AND voluntary euthanasia | National parliament act |
| New Jersey (USA) | Assisted suicide | State legislature act |
| New Mexico (USA) | Assisted suicide | State legislature act |
| New South Wales (Australia) | Assisted suicide AND voluntary euthanasia | State parliament act |
| New Zealand | Assisted suicide AND voluntary euthanasia | National parliament act |
| Oregon (USA) | Assisted suicide | State legislature act |
| Portugal | Assisted suicide AND voluntary euthanasia | National parliament act |
| Queensland (Australia) | Assisted suicide AND voluntary euthanasia | State parliament act |
| South Australia (Australia) | Assisted suicide AND voluntary euthanasia | State parliament act |
| Spain | Assisted suicide AND voluntary euthanasia | National parliament act |
| Switzerland | Assisted suicide | Federal criminal code |
| Tasmania (Australia) | Assisted suicide AND voluntary euthanasia | State parliament act |
| Vermont (USA) | Assisted suicide | State legislature act |
| Victoria (Australia) | Assisted suicide AND voluntary euthanasia | State parliament act |
| Washington (USA) | Assisted suicide | State legislature act |
| Western Australia (Australia) | Assisted suicide AND voluntary euthanasia | State parliament act |
